# Short-Term Effects of Hospital Schooling on the Resilience of Hospitalised Children

**DOI:** 10.5334/cie.172

**Published:** 2025-07-15

**Authors:** Ana Padillo-Andicoberry, Francisco de Asís Díaz-Beato, Encarnación Sánchez-Lissen, Clara Romero-Pérez

**Affiliations:** 1 University of Seville, Seville, Spain; 2 International University Isabel I of Castilla, Burgos, Spain; 3 University Hospital Virgen del Rocío, Spain

**Keywords:** children’s disease, hospital school, resilience, school provision

## Abstract

Stresses related to illness, hospitalisation, and the disruption of educational activities and daily routines often have a negative impact on children, with panic situations or anxiety states being the most frequent manifestations. This study explores whether participation in routine hospital school activities is associated with short-term changes in children’s resilience during hospitalisation. The present study aimed to examine the resilience dimensions upon entering and leaving the hospital school and to analyse whether the curriculum-based activities and other parameters related to disease and hospitalisation could influence children’s resilience evolution. A prospective study was conducted with 52 children (31 girls and 21 boys) aged nine to 14 years, who attended the hospital school. The average attendance was one week. The School Resilience Scale for children, which includes five dimensions, was used at admission to the hospital school and before hospital discharge. An adapted visual Likert scale was repeatedly applied after school provision to assess children’s satisfaction with the curriculum-based activities. Upon admission, the resilience percentile was 50.19, improving to 63.40 before discharge (*p* = 0.022). This improvement was higher in children who attended more than three days of school (*p* = 0.014). Enjoyment of activities (*p* = 0.029) and the perception that school lessons helped the children not to worry about illness (*p =* 0.045) were the only variables associated with the SRS improvement. The observed results suggest that educational activities provided in the hospital school during the evaluated period can positively enhance resilience in hospitalised children.

Hospital settings can make children prone to negative experiences that might result in an increased emotional vulnerability (Lulgjuraj & Maneval, 2021). The uncertainty that paediatric patients often undergo during their hospitalisation can lead to emotional dysfunction, expressed as sustained stress, as well as panic or anxiety states (Demetriou et al., 2024; Ko et al., 2022). For these reasons, different authors consider that enhancing children’s resilience in the hospital context can counteract the emotional impact of their illness and hospitalisation (Coyne & Kirwam, 2012; Rosenberg et al., 2019; Shah et al., 2020).

## Conceptualising Resilience

Resilience is broadly defined as the outcome of a functional or positive adaptation process in the context of adversity (Cahill, 2022; Mesman et al., 2021; Van der Laan et al., 2023). From a theoretical point of view, four central approaches can be recognised in the conceptualisation of resilience: resilience as adaptability, resilience as capacity, resilience as a combination of risk and protective factors, and resilience as a process (García del Castillo et al., 2016).

In the framework of this study, which focuses on hospitalised children, the theoretical approach is based on the resilience construct as inner strength or fortitude, drawing on the theories developed by Grotberg (2006), Kuranova et al. (2021), Schultze-Lutter et al. (2016), and Suriá Martínez (2017). These theories encompass social and personal competence, as well as acceptance of oneself and emotional self-regulation. The focus of resilience has often been on stress-coping mechanisms and problem-solving skills in adverse situations. In clinical and educational frameworks, resilience competencies become increasingly important in sick and/or hospitalised children due to the influence they exert on mental health. In this reality, resilience is linked to overall psychological health. In the case of hospitalised children, it can be promoted and encouraged by providing stimulating environments where they can safely develop individual competencies (Grotberg, 1995, 2003; Henderson & Milstein, 2003; Vanistendael & Lecomte, 2002). In the context of Spanish-speaking countries, one of the most widespread theoretical models is the one by Grotberg (Fernández-Ramírez, 2011; González Arratia et al., 2009; Muñoz-Garrido, 2016; Serradas, 2016). This model assumes that resilience is a result of the interaction of three factors: inner strength (I am); external support (I have), and interpersonal and conflict resolution skills (I can). In a complementary way, Saavedra (2003), based on Grotberg’s model (1995, 2003), further expanded this notion by considering that resilience is the result of the interaction of four factors: starting conditions, self-perception, interpretation of the problem, and active or resilient response to adversity. The latter model allows for a proper differentiation of the areas of resilience according to how developed they are, leading to psychoeducational interventions through the construction of profiles and the delimitation of objectives. As a result of the interaction of both models, Saavedra & Castro (2009) created and validated the School Resilience Scale (SRS) for the school population. The psychometric properties of this scale are robust in terms of validity and reliability.

## Resilience in Hospitalised Children

Even though hospital environments and children’s hospitalisation can lead to a deterioration of their resilience capacities, studies focusing on the resilience of hospitalised children are scarce (Gajardo et al., 2024; García-Parra et al., 2021). In addition, the same lack of research can be observed in studies related to the instruments used for the assessment of resilience in hospital contexts (Quevedo Mojica & De la Peña, 2018). There is consensus that both individual aspects, as well as the family and social environment, play a role in enhancing resilience in hospitalised children (Grotberg, 2003; Seko et al., 2020; Storch & Reigada, 2024). Hence, it is essential to conduct a comprehensive assessment of their resilience capacities, using tools such as those presented by Saavedra & Castro (2009) that address all dimensions and resources.

Although, as previously mentioned, several factors can influence the resilience of hospitalised children, this study focuses on the curriculum-based activities that take place in hospital schools and their short-term impact on children’s resilience in the hospital context.

## Hospital Schools as Resilience-Promoting Spaces

On the one hand, hospital schools serve a pedagogical function that make them spaces for inclusive education that allows sick hospitalised children to continue their studies and return to school after their recovery (Bastidas-Rivera et al., 2023; Ciucci et al., 2024; Gútiez-Cuevas & Muñoz-Garrido, 2021; Lizasoáin, 2021; Ludgério et al., 2023). Therefore, the activities conducted should be tailored to the possibilities of each child according to their health status (Castro Ibáñez, 2023; García-Parra et al., 2021). On the other hand, they also have a therapeutic role, offering emotional support to hospitalised children. In addition, teachers in hospital schools, with their specialised training, experience, and educational style, create an enriching environment for hospitalised children, helping them to overcome the negative impact of the illness itself and hospitalisation (Ávalos & Fernández, 2021; Benigno & Fante, 2020; Hen & Gilan-Shochat, 2022; Keehan, 2021; McNamara, 2024). Furthermore, several professional development programs have been proposed (Małkowska-Szkutnik et al., 2021; Maor et al., 2020; Mombaers & Donche, 2020), suggesting the development of teachers’ emotional competence to handle complex situations, such as those encountered in the hospital setting (McNamara, 2024).

Currently, very little is known about the impact of the school lessons taught in hospital schools on the resilience of the children admitted. The existing literature published in English and Spanish consists of only two studies. The first is a descriptive study in which the authors present the ordinary activities they carry out and conclude that these activities could theoretically promote child resilience in hospital schools. However, they do not conduct a specific analysis of resilience (Muñoz-Garrido, 2016). The second, recently published, is an experimental study, conducted by Ciucci et al. (2024), which evaluated the impact of the hospital-based school lessons that were implemented in an Italian hospital school for the reduction of negative emotions, distress and pain in hospitalised children. Their evaluations yielded favourable results. However, again, resilience was not specifically studied. Therefore, there is a need to understand the possible relationships between the hospital school provision and their impact on the improvement of resilience in hospitalised school-age children. The evidence of such an impact is not easy to determine because resilience is also understood as a dynamic and evolving process. Therefore, one of the possible limitations of studies evaluating intervention effectiveness is related to the time needed to achieve significant results. Although most studies that evaluate resilience improvement programs in children include medium and long-term actions over several weeks (Noyola et al., 2024; Ridings et al., 2019), other studies, such as the one conducted by Roque-López et al. (2021), provide evidence of positive results using short-term, one-week intervention programmes. This is important because, unlike regular schools, hospital schools must adapt their activities to the time the children spend in the hospital. This time can vary from short stays of less than a week to long stays of weeks or months (Di Padova et al., 2024).

To our knowledge, no studies have explored the relationship between hospital-based school lessons taught in hospital schools during a short period and the resilience of hospitalised children. The present study aims to address this gap of knowledge in the available literature.

## Purpose of the Study

This study explores whether participation in routine hospital school activities is associated with short-term changes in children’s resilience during hospitalisation. The present study aimed to answer the following research question: Does participation in curriculum-based activities during a short hospital stay contribute to measurable changes in the resilience of hospitalised children?

By addressing this question, the objectives of the study were as follows:

1. To evaluate the overall resilience and its dimensions in a cohort of hospitalised children, analysing whether certain parameters related to the disease, such as previous admissions or the type of disease, play a role in the baseline status of resilience.
2. To assess the degree of children’s satisfaction with the hospital school provision.
3. To evaluate the changes observed in resilience throughout the hospital school provision.
4. To analyse whether the hospital school provision plays a role in the evolution of resilience, considering other parameters related to disease and hospitalisation.

## Method

### Participants

To achieve the stated objectives, a prospective pre-post study with an intervention group (Alessandri et al., 2017; Jordan et al., 2009) was conducted at the hospital school of a University Hospital. Informed consent to participate in the study was obtained from the parents. The study was approved by the Ethics Committee of the University Hospital on December 2, 2022 (reference code: 2002-N-22).

For our study, the exclusion criteria were participants younger than nine years old or older than 14 years old; children with special needs; lack of parental authorization; the patient’s unwillingness due to their emotional state; and the inability to attend the hospital school for medical reasons.

**Figure 1:**
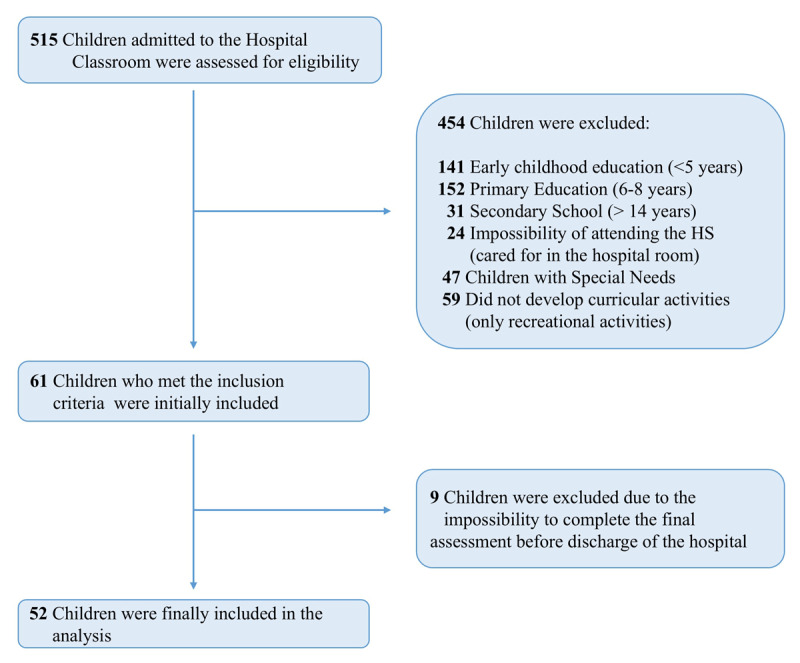
Flow chart of the study. *Note*. The figure shows the complete participant flow chart of the study.

As shown in Figure 1, in this study, 52 children admitted to the University Hospital Virgen del Rocío (Seville, Spain) attending the hospital school were included (31 girls and 21 boys).

As shown in Table 1, the sociodemographic characteristics of the participants.

**Table 1:** Sociodemographic Characteristics of the Participants.


VARIABLES		AGE (YEARS) [MEAN (*SD*)]	FIRST ADMISSION [*n*(%)]	READMISSIONS [*n* (%)]	NUMBER OF READMISSIONS [MEAN (*SD*)]	HOSPITAL ATTENDANCE (DAYS) [MEAN (*SD*)]	ACUTE DISEASE [*n*(%)]	CHRONIC DISEASE [*n*(%)]

Overall		11.32 (1.90)	38 (73%)	14 (27%)	4.63 (4.30)	9.93 (9.60)	41 (78.6)	11 (21.2)

Gender	Female (*n =* 31)	11.51 (1.99)	22 (71%)	9 (29%)	2.56 (1.50)	9.20 (9.38)	25 (80.6)	6 (19.4)

	Male (*n* = 21)	11.00 (2)	16 (76%)	5 (24%)	5.60 (8.11)	10.67 (10)	16 (76.2)	5 (23.8)

	*p* value	0.97	0.68		0.28	0.59	0.74	

	Mean difference (95% *CI*)	–0.49 (–21.0–20.7)	NA	NA	3.04 (–2.84–8.93)	–1.46 (–4.95–6.08)	NA	NA

	Power (%) (*Z* value/*SE*)	NA	13.36 (0.422/0.123)	5.55 (–0.422/0.136)	NA	NA		


*Note*. NA = not applicable; *SD* = standard deviation; *SE* = standard error; *CI* = confidence interval.

While it was the first hospital admission for 38 children (73%), the remaining 14 (27%) had previously been admitted to a medical facility, with a mean of 4.63 admissions (*SD*: 4.30). The mean hospital stay was 9.93 days (*SD*: 9.60).

### Hospital School Provision

#### Pedagogical objectives

Three teachers and a teaching assistant developed the hospital school programme of the University Hospital Virgen del Rocío (Seville, Spain). The programme uses curriculum-based activities which are related to the child. These tasks include three areas (Mathematics, Spanish Language, and Science) and are tailored to the children’s age and curricular level, as well as to their physical and emotional state. Children are also offered digital, playful (board games, body expression and dramatizations) and artistic activities. Additionally, the programme also involves projects with social organizations.

#### Coordination with families and health care professionals

First, to build relationships with the newly admitted child and their family, teachers provide the corresponding school service. Engaging with the family is a key aspect of the teachers’ intervention and served as a starting point. Families do not attend the hospital school in order to encourage children’s autonomy and collaboration with the teachers without interference. Finally, hospital teachers coordinate the school provision with other hospital professionals and regular schools to ensure a consistent educational approach.

### Procedure

#### Participants Integration at the Hospital School and Initial Evaluation

Once children are admitted to the hospital, the hospital school coordinators receive information about the child’s possible incorporation into the school. Upon admission, students fill out a form to collect personal data and information about their educational level and the activities they do at their school. This allows teachers to prepare the school lessons that are developed in the following days.

#### Protocol of data collection

On the first day of attending the hospital school, the students complete the resilience assessment questionnaire, as described in the instruments section. This scale is used upon admission and again before discharge from the hospital. Educational lessons are not introduced on the first day of attendance. On the second day of school attendance, the students are integrated into the regular activities of the hospital school. The daily duration of school attendance is 3 hours, during which, students engage in educational activities. Furthermore, an adapted visual scale is used to assess enjoyment of the curriculum-based lessons developed during the hospital school attendance period. This assessment begins on the second day of school attendance and is conducted every two days until discharge. The average value is used for analysis. The following section offers more details on the employed scale.

**Figure 2:**
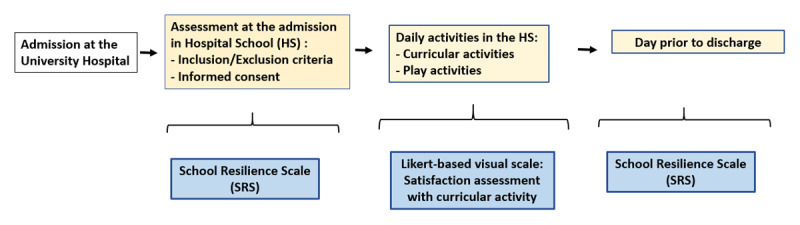
Study Protocol. *Note*. Figure 2 shows a summary of the study protocol.

### Measures

#### Resilience Assessment

The SRS for children from nine to 14 years old (Saavedra & Castro, 2009) was used. This is a scale, validated by the authors, with a Cronbach’s alpha reliability of 0.88 (Saavedra & Castro, 2009). The SRS includes 27 questions ranging from one point (strongly disagree) to five points (strongly agree). The overall test score ranges from nine to 27 points. Five dimensions are evaluated to define specific resilience capabilities: 1) Identity and self-esteem (I am), including 9 questions (from 9–15 points); 2) Environment: perception of networks and models (I have), including 9 questions (from 9–15 points); 3) Learning capacity (I can), including 9 questions (from 9–15 points); 4) Internal resources, referring to the characteristics that have a more personal dependence on the subject, more structural capabilities, including 13 questions (from 13–65 points) and 5) External resources, referring to interactional characteristics that the subject establishes with its environment, including 14 questions (from 14–70 points). According to the protocol, the values obtained in each dimension are adjusted to percentiles. In the case of the overall SRS, the percentiles are adjusted by gender. The interpretation of the percentile scores is as follows (Saavedra & Castro, 2009): low level of resilience capacity: percentiles 1 to 25; medium level of resilience capacity: percentiles 26 to 74; and high level of resilience capacity: percentiles 75 to 99.

#### Assessment of the Enjoyment of the Hospital School Provision

An adapted visual scale based on the Likert proposal with five options and a score ranging from one to five was used for the evaluation of enjoyment with curriculum-based activities (Salam & Ismail 2015). The proposed scale was used on the basis that student satisfaction and enjoyment are usually measured using adapted researcher-designed questionnaires based on a visual Likert scale (Bedard et al., 2019; Mavilidi et al., 2017). Two dimensions were evaluated for each school lesson taught (Spanish language, Mathematics, and Science): “I enjoyed learning the lesson” and “This lesson helped me not to worry about my stay in the hospital”.

### Data Analysis

Data analysis was performed in the overall sample of 52 children who attended the hospital school. The normal distribution of the values was achieved. The quantitative variables are expressed as mean and standard deviation (*SD*).

The study’s first aim, which focuses on determining whether parameters such as gender, previous admissions, or type of disease are associated with resilience at admission, was achieved by conducting an unpaired Student’s T-test.

Additionally, another unpaired Student’s T-test was employed to tackle the second objective of the study, related to the possible association between these parameters and the satisfaction values registered with the Likert scale.

To analyse the variations in the dimensions of resilience during the hospital school attendance included in the third objective, a paired Student’s T-test was used to compare the values of each dimension upon admission and discharge. The mean difference and its 95% confidence interval (CI) were measured as magnitude.

To achieve the fourth objective, a mixed ANOVA, controlling for the effect of the number of school days attended, was employed to achieve differences between analysed covariates and SRS determinations. This test considered the two SRS measurements at different times for each subject (SRS at admission and discharge), including the variable days of hospital school attendance as a between-subjects factor. For this purpose, the variable days of hospital school attendance during hospital admission was dichotomized according to its median value.

In addition, a univariate linear regression analysis was performed to determine predicting factors for the variation in the SRS between admission and discharge according to the values of the different covariates. For this analysis, the variable ΔOverall SRS was employed as the dependent variable for the linear regression analysis (ΔOverall SRS = discharge SRS – admission SRS). Beta values (β) and their 95% Confidence Interval (CI) were calculated for each variable to get a measure of the estimated change in SRS scores between admission and discharge for each change in the predictor variables.

Furthermore, to analyse the correlation between continuous variables, Pearson correlation coefficients were also calculated. Results were considered statistically significant when the *p* (probability) value was <0.05. Statistical analysis was performed using SPSS 25 for Windows (SPSS Inc, Chicago, IL).

## Results

### Baseline SRS Assessment at Admission in the Hospital School

The global resilience of the children measured with the SRS upon admission to the hospital school was at the 50.19 percentile (*SD*:30.5), without differences between gender.

Table 2 shows the results obtained related to the first objective of the study, which focused on the evaluation of the potential impact that previous admissions to the hospital and type of disease may have on the baseline resilience of the children upon entrance to the hospital school. A comparison of all SRS dimensions according to these variables was performed. There were no significant differences between groups. Moreover, no correlation was found between the number of previous hospitalisations and the SRS upon admission to the hospital school (*r* = 0.27; *p* = 0.36).

**Table 2:** Assessment of School Resilience Scale (SRS) upon admission to hospital school. Analysis of risk factors associated with the disease that could affect resilience.


VARIABLES		OVERALL SRS	IDENTITY	ENVIRONMENT	LEARNING	INTERNAL RESOURCES	EXTERNAL RESOURCES

Gender	Girl	51.26 (31.10)	52.70 (28.99)	55.19 (31.72)	50.51 (31.98)	46.29 (30.93)	50.97 (27.81)

	Boy	49.12 (29.91)	50.11 (30.51)	49.17 (34.51)	52.40 (31.32)	42.11 (27.51)	52.14 (29.85)

	*p-value*	0.674	0.972	0.523	0.954	0.361	0.965

	Mean difference (95% *CI*)	–4.14 (–24.0–15.7)	–0.32 (–18.8–18.2)	–6.77 (–28.0–14.5)	–0.59 (–21.1–19.9)	–8.7 (–27.9–10.4)	–0.46 (–21.5–20.6)

Admissions	First	52.15 (30.4)	54.13 (31.82)	51.33 (34.43)	58.11 (33.81)	46.12 (26.84)	51.13 (23.83)

	Readmission	48.23 (30.6)	48.12 (27.68)	52 (31.80)	48.15 (29.49)	42.11 (31.60)	51 (33.83)

	*p-value*	0.599	0.301	0.853	0.375	0.488	0.707

	Mean difference (95% *CI*)	5.58 (–15.7–26.9)	7.29 (–9.6–30.1)	–2.12 (–25.1–20.9)	9.62 (–12.1–31.3)	4.12 (–13.5–27.7)	0.23 (–18.4–26.8)

Type of disease	Acute	52.00 (30.65)	59.85 (28.57)	54.83 (32.63)	57.67 (32.82)	51.50 (29.63)	60.83 (32.86)

	Chronic	41.00 (27.97)	41.00 (28.17)	44.00 (32.73)	46.00 (25.25)	35.00 (26.67)	51.50 (30.01)

	*p-value*	0.210	0.074	0.369	0.312	0.127	0.432

	Mean difference (95% *CI*)	14.00 (–8.2–36.2)	18.85 (–1.9–39.6)	10.83 (–13.3–35.0)	11.67 (–11.4–34.7)	16.50 (–4.9–37.9)	9.33 (–14.5–33.1)


*Note*. Data are expressed as the mean of the obtained adjusted percentile and standard deviation (*SD*). There were no statistical differences between values. *CI* = Confidence Interval.

### Process Outcomes: Satisfaction and Enjoyment of the Lessons Taught in the HS

The average hospital school attendance was 5.51 days (*SD*: 6.21). Most children were able to carry out established curricular-based activities, and in only one case, a curricular adaptation was needed. Regarding the assessment of school activities measured using a Likert-based visual scale, the students valued the activities positively, with 74% over the maximum (3.7 out of 5 points; *SD*: 0.63). Furthermore, they also considered that it helped them cope with the hospital experience at a rate of 70.9% of the maximum (3.54 out of 5 points; *SD*: 0.58). Comparisons according to gender, previous admissions, and the type of disease as proposed in the second objective are presented in Table 3.

**Table 3:** Assessment of satisfaction with school provision according to the adapted Likert-based visual scale.


VARIABLES		ENJOYMENT	USEFULNESS

Gender	Girl	3.47 (0.64)	3.29 (0.64)

	Boy	3.94 (0.61)	3.80 (0.52)

	*p* value	0.22	0.23

	Mean difference (95% *CI*)	0.47 (–0.34–1.28)	0.51 (–0.41–1.42)

Admissions	First	3.72 (0.75)	3.83 (0.47)

	Readmission	3.76 (0.64)	3.33 (0.76)

	*p* value	0.91	0.28

	Mean difference (95% *CI*)	–0.04 (–1.01–0.92)	0.5 (–0.55–1.55)

Type of disease	Acute	3.80 (0.69)	3.64 (0.64)

	Chronic	3.44 (0.51)	3.44 (0.63)

	*p* value	0.44	0.68

	Mean difference (95% *CI*)	0.35 (–0.63–1.33)	0.19 (–0.87–1.26)


*Note*. Enjoyment means: “I enjoyed learning the lesson”. Usefulness means: “This lesson helped me not to worry about my stay in the hospital”. Values are expressed as mean *(SD*). *CI* = Confidence interval.

**Figure 3:**
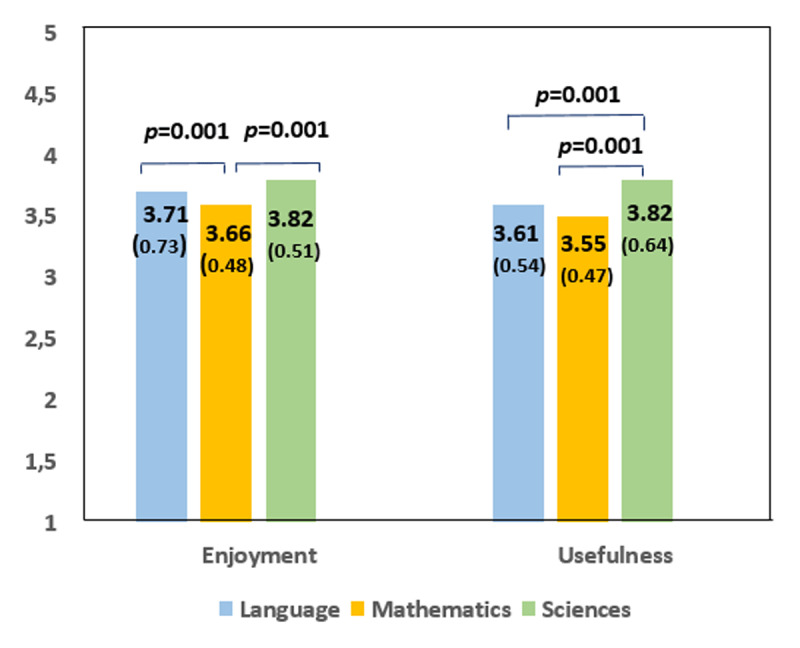
Assessment of perceived satisfaction and usefulness with school lessons according to the adapted Likert visual scale. *Note*. Enjoyment: “I enjoyed learning the lesson”. Usefulness: “This lesson helped me not to worry about my stay in the hospital”. Values are expressed as mean (*SD*).

In addition, as shown in Figure 3, significant differences were observed when comparing the subjects of the assessed lessons. Activities related to Science and the Spanish language generated greater satisfaction than Mathematics-related activities (*p* = 0.001). Science-related activities were the most effective in reducing worries about illness and hospitalisation (*p* = 0.001) (Figure 3). No significant differences were observed according to gender.

Furthermore, there was a correlation between children’s lesson enjoyment at the hospital school and the perception that this activity was useful in reducing their concern about the hospital experience *(r* = 0.62; *p* = 0.003). This was reproduced in the three curriculum-based activities evaluated. However, no correlation was found between school lesson assessments and duration of hospital school attendance (*r* = 0.33; *p* = 0.29 in “enjoyment” and *r* = 0.27; *p* = 0.48 in “usefulness”).

### Pre-Post Intervention Evaluation of SRS

Regarding the third objective, it was observed that prior to discharge from the hospital, the overall resilience evaluated at the hospital school increased significantly when compared with the initial resilience assessment upon admission (from percentile 50.19 [*SD*: 30.5] to percentile 63.40 [*SD*: 35.12]; *p* = 0.022). This improvement was mainly related to an increase in resilience skills associated with the dimensions of identity (*p* = 0.027) and internal resources (*p* = 0.011), as shown in Table 4.

**Table 4:** Comparison of the School Resilience Scale (SRS) assessment upon admission to the hospital school and before the hospital discharge.


VARIABLES	OVERALL SRS	IDENTITY	ENVIRONMENT	LEARNING	INTERNAL RESOURCES	EXTERNAL RESOURCES

Admission	50.19 (30.5)	51.44 (29.75)	51.66 (33.11)	51.45 (31.61)	44.20 (29.22)	51.55 (28.83)

Discharge	63.40 (35.12)	62.88 (35.81)	66.00 (33.26)	59.60 (34.3)	60.80 (35.35)	56.40 (33.48)

*p-value*	**0.022**	**0.027**	0.061	0.432	**0.011**	0.116

Mean difference(95% *CI*)	–12.8(–23.6 – –2.0)	–11.5(–21.7 – –1.4)	–14.2(–27.0–0.6)	–5.8(–13.6–6.0)	–16.4(–28.7 ––4.1)	–3.0(–18.1–2.1)


*Note*. Data are expressed as the mean of obtained adjusted percentile and standard deviation (*SD*). *CI* = confidence interval. In bold *p*-value <0 .05.

To achieve the fourth objective of evaluating whether the educational activities developed in the hospital school and other coexisting factors influence the evolution of resilience throughout the hospital school experience, both an ANOVA test and a linear regression were conducted.

The mixed ANOVA indicated that children who attended the hospital school for three or more days showed a significant improvement in SRS values from admission to discharge with significant associations within-subjects (p = 0.035) and between-subjects (0.014) as shown in Table 5.

**Table 5:** SRS means upon admission and discharge by groups of length of school attendance.


SCHOOL ATTENDANCE	SRS AT ADMISSION [MEAN (SD)]	SRS AT DISCHARGE [MEAN (SD)]	BETWEEN-SUBJECTS *p*-VALUE

<3 days	77 (18)	76 (30)	0.014

>3 days	36 (28)	57 (37)	

Within-subjects p-value	0.035		


*Note*. Within-subjects and between-subjects p-values of mixed ANOVA tests are shown.

After controlling for school attendance on the mixed ANOVA model, there were no differences in SRS within-subjects or between-subjects by gender, age, type of disease, readmissions, or Likert scale values (see Table 6).

**Table 6:** Mixed ANOVA tests with SRS values upon admission and discharge as dependent variables.


VARIABLE	*p*-VALUE	

	Within-subjects	Between-subjects

Gender	0.939	0.812

Age (<11 years vs >11 years)	0.988	0.990

Chronic disease	0.462	0.774

Readmission	0.534	0.460

Likert – Enjoyment	0.577	0.108

Likert – Usefulness	0.656	0.779


*Note*. The associations of the different variables were measured within-subjects and between-subjects, considering the between-subjects factor “school attendance” to control the interaction of this variable.

The results of the linear regression study conducted with all the considered variables showed that children who attended the hospital school for three or more days increased SRS by 22.6 points compared to those who went less than three days (95% CI 1.8–43.5, *p* = 0.035). There were also significant associations with both the Likert scale scores: for each point of the Likert–Enjoyment scale, the SRS score increased by 32.2 points (95% CI 4.1–60.2, *p* = 0.029) and for each point of Likert-Usefulness scale, it improved 21.8 points (95% CI, 0.7–42.8) (see Table 7).

**Table 7:** Univariate linear regression analysis for ΔOverall SRS.


VARIABLE	*p* VALUE	*β* (*CI* 95%)

Gender	0.489	–7.4 (–29.3–14.4)

Age (year)	0.724	0.9 (–4.5–6.4)

Readmission	0.954	–0.7 (–23.2–24.5)

Chronic disease	0,838	–2.6 (–28.4–23.3)

Hospitalisation (days)	0.365	–0.551 (–0.684–1.786)

HS Attendance > 3 days	**0.035**	22.6 (1.8–43.5)

Likert – Enjoyment	**0.029**	32.2 (4.1–60.2)

Likert – Usefulness	**0.045**	21.8 (0.7–42.8)


*Note*. HS = Hospital School. *CI* = confidence interval. In bold *p* value < 0.05.

The continuous variables with statistically significant results in the linear regression analysis (“enjoyment with the lessons taught” and “the lessons taught helped me not to worry about the disease”) were then correlated with the Δ SRS in each SRS dimension. As a result of this analysis, the enjoyment of the curricular activities was associated with an improvement in SRS for the learning dimension (*r* = 0.58; *p* = 0.05) and with the SRS for the external resources dimension (*r* = 0.68; *p* = 0.020). In addition, the perception that the lessons taught helped the children not to worry about the disease showed strong correlations with both SRS for the identity dimension (*r* = 0.88; *p* = 0.008) and for the internal resources dimension (*r* = 0.89; *p* = 0.007).

## Discussion

The main aim of the present study was to measure potential changes in resilience associated with participation in hospital school activities. The study shows that hospital school provision for a short period of time helps children to not worry about illness and hospitalisation. These positive perceptions during the hospital school experience were associated with increased resilience, especially reinforcing the self-esteem and inner resources of the hospitalised children.

For these children, hospitalisation means leaving their home, parents, and siblings. It also disrupts their daily routines at school as well as their playtime with friends. In addition, the emotional impact of the disease and hospitalisation has a multifactorial origin. On the one hand, the severity of the disease and whether the illness is an acute or chronic disease could significantly impact the emotional status of the children (Fortier & Kain, 2015). On the other hand, the family environment plays a crucial role in hospitalised children (Grotberg, 2003; Seko et al., 2020). Furthermore, children usually associate hospital environments with pain and discomfort as a direct consequence of the tests and treatments that they undergo during their recovery. All this generates fear and insecurity in the face of the unknown (Burns-Nader & Hernández-Reif, 2016; Koukourikos et al., 2015).

The impact of these disorders on minors may depend on children’s resilience skills. Those with high levels of resilience may develop a less traumatic experience (Denckla et al., 2020; Garcia-Parra et al., 2021; Kuranova et al., 2021; Lee et al., 2021). Therefore, in order to improve clinical and emotional outcomes, it is important to assess the state of resilience skills in hospitalised children. The theoretical framework of this study is based on the concept of resilience as inner strength. It draws on the theories developed by Grotberg (2006), Kuranova et al. (2021), Schultze-Lutter et al. (2016), and Suriá Martínez (2017), that encompass several dimensions, such as the environment, self-esteem, and the ability to develop learning skills. In this study, the SRS (Saavedra & Castro, 2009) was used to assess resilience capabilities in hospitalised children. As previously proposed by Grotberg’s model (1995, 2003), this research also showed that hospitalised children exhibited a resilient attitude due to their inner strength and interpersonal and conflict resolution skills.

Responding to the first objective set out in the study, upon entering the hospital school, the participants in the study showed overall levels of resilience around the 50th percentile. This indicates a medium level of resilience, according to the qualitative equivalences of interpretation (percentile range from 26 to 74). Additionally, the resilience skills were very homogeneous across the five evaluated dimensions. Research on resilience in hospitalised children is scarce, and not all studies include the necessary dimensions for a correct assessment of these children (Grotberg, 2003; Henderson & Milstein, 2003). In a study of 40 children with chronic kidney disease, Xavier et al. (2024) found that these children exhibited medium-high resilience. While the results were similar to those of the present study, comparisons cannot be made given that they are related to children with chronic diseases but not hospitalised.

In the context of hospitalisations, authors like Wilcox (2018) noted that children with more than one episode of hospitalisation have a reduced ability to cope with the stress associated with the new illness, leading to lower resilience. However, our study found no correlation between the number of readmissions and the SRS values observed upon admission to the hospital school. One possible explanation for this is that attending a hospital school may have provided a protective effect on the resilience capabilities of previously hospitalised children (Caleffi et al., 2016). Our study also examined whether the presence of an acute or chronic disease could influence children’s emotional status (Coventry et al, 2025), but this variable did not show any influence on SRS values upon admission. In addition, while several studies have explored the positive impact of play-based activities on children’s emotional well-being during their hospitalisation period (de Jong et al., 2020), very few have assessed the potential role of curriculum-based activities on children’s resilience capacities in hospital settings (García-Parra et al., 2021). In the hospital school programme presented in this study, we combined educational games, as well as artistic, digital, playful, and curriculum-based activities, following the criteria of standard practice. However, only the latter were included in the study to assess their potential role in resilience capacities.

For the second aim of this study, two main questions were asked and evaluated using the adapted visual Likert scale. One of them aimed to assess children’s satisfaction with the activities (enjoyment), while the other intended to evaluate how useful the lessons were at reducing children’s worry about their medical condition (usefulness). As expected, the more the children enjoyed the lessons, the less they worried about the hospital experience. No differences in the perception of curriculum-based activities were observed between children who had been admitted previously and those who were admitted for the first time.

For our third objective, we also evaluated the evolution of resilience upon discharge from the children’s hospital. One of the main findings of the study was that after attending the hospital school, the overall resilience of the students increased significantly, reaching a higher level. This improvement primarily stemmed from strengthening resilience dimensions related to identity and internal resources. Currently, while there is considerable theorising, very little is known about the impact of hospital school activities on enhancing the resilience of incoming children. This may be because most studies evaluating resilience-enhancing programs for children focus on medium- and long-term actions over several weeks (Noyola et al., 2024; Ridings et al., 2019), overlooking the effects of short-term activities. However, a study by Roque-López et al. (2021) addressed this issue, showing improvements in resilience with the implementation of short one-week intervention programs, similar to those developed in this study. According to Grotberg (2006), resilience is activated when adversity is experienced, and hospitalisation can be seen as an unfavourable experience for the child, occurring abruptly and unexpectedly, which triggers the activation of resilience. This may explain the results shown in the present study.

Based on these results, one might then ask what role the hospital-based school lessons play in the observed reinforcement of children’s resilience. This was our fourth objective in which, in addition to assessing hospital school activities as a possible enhancer of resilience, we also considered other factors related to the disease or hospitalisation that could modulate it. In this regard, as observed in the admission assessment, neither the type of disease (acute or chronic) nor the number of previous admissions correlated with SRS evolution during hospital school attendance. However, given that to our knowledge there are no references in other studies, no comparative analysis can be performed. Moreover, further lines of work may contemplate other factors that could modulate the emotional state of these children but have not been analysed in our study and which are different to those related to hospital schools, such as the family environment (Isokääntä et al., 2019) or the hospital environment itself (Kindervaag et al., 2024).

Nevertheless, the potential role of hospital schools in enhancing resilience skills has been specifically confirmed in this study, with the observed association between students’ positive perceptions of the school lessons provided (both in terms of enjoyment and how useful they were in reducing their worry) and an improvement in general resilience skills. Moreover, hospital lessons can enhance the quality of children’s lives (Ciucci et al., 2024). Specifically, the perception that the school provision helped children not to worry about their illness was notably associated with a strengthening of the SRS dimensions of children’s self-identity and inner resources. The relevance of this finding lies in the fact that the hospital school experience reinforced personal strengths and self-esteem, which are key goals for enhancing resilience in children (Alvord & Grados, 2005). This is supported by the work of O’Connor et al. (2017), who emphasize the importance of supporting hospitalised children’s emotions and resilience through active and passive distractions. Among the active distraction activities, those that involve cognitive activities that require selective attention and problem-solving play a specific role in the context of the hospital school.

Interestingly, in our study, children who attended the hospital school for more than three days exhibited a greater increase in resilience values than those who were there for fewer days. This indicates that despite the negative effects that hospitalisation may have (Rokach, 2016), as the days go by, the activities carried out in the hospital school provide progressive reinforcement to children. Moreover, although our findings suggest that the curriculum-based activities helped strengthen children’s resilience, one could also think that the other playful activities played some positive role in children’s resilience. Although no studies evaluating this aspect have been found in the available literature, Anggraini et al. (2022) showed in a literature review that educational play has a positive effect on children’s emotional state by reducing their anxiety. This highlights the importance of integrating educational programmes into the concept of hospital pedagogy (Lizasoain, 2021).

## Conclusion

The present study aimed to answer the research question related to whether the educational activities conducted during a short period of time in a hospital school could influence the global and dimensional resilience of hospitalised children. Our findings support the idea that hospital school lessons are useful, not only for educational purposes, but also as a tool to enhance children’s adaptation to adverse situations.

In response to our first objective, related to the assessment of resilience upon arrival to the hospital school and the factors that can modulate it, we observed that sick children admitted to hospitals have resilience skills that are in the middle percentiles. Additionally, children with previous admissions displayed lower resilience skills upon admission to the hospital school.

Once in the hospital school, children evaluated the lessons positively in terms of both enjoyment and whether they helped them not to worry about their illness and hospitalisation.

By the time they were discharged from the hospital, the children’s dimensions of resilience were significantly strengthened. When factors associated with this improvement were evaluated, only the positive perception of educational activities achieved a significant correlation. Furthermore, despite the negative effects that hospitalisation may have, children who attended the hospital school for more than 3 days exhibited a greater increase in the dimensions of resilience than those who went for less time.

Although several factors may be involved in improving resilience, our study showed that the school lessons contributed to enhancing learning skills, which was to be expected, and, perhaps more importantly, to strengthening children’s inner resources and self-esteem.

### Implications for Practice

The results obtained in our study may be of great interest to teachers at hospital schools. The relevance of our findings lies in the fact that these professionals need children’s health-related information to implement specific programmes for the promotion of resilience and prevention of emotional problems in school settings. This can be accomplished through screening systems (Casares et al., 2024). Our results highlight the teachers’ need to assess children’s resilience upon hospital admission. This would allow special attention to be given to the most vulnerable minors, such as children with previous admission, who displayed lower resilience skills in our study.

Furthermore, it would also be useful and straightforward to evaluate the degree of acceptance of the activities carried out in the hospital school. Based on our results, this would allow professionals to detect those children who may not be evolving positively in their emotional state. This appraisal would facilitate information for teams of psychologists so that they can assess specific needs and provide more targeted actions.

Finally, the evaluation of the resilience data upon discharge from the hospital may help examine children’s adjustment to their regular schools and develop precise actions for a smoother transition.

All these suggestions imply the creation of motivated multidisciplinary teams, highly prepared at scientific, psychological, social, and pedagogical levels (Muñoz-Garrido, 2016) to evaluate not only resilience but also how children perceive the school lessons. These programmes, as Capurso et al. (2020) suggest, should tailor hospital school activities to their social context and cultures.

### Implications for Further Research

In the present study, the duration of the hospital school attendance was associated with positive values of resilience upon discharge from the hospital. Given that only curriculum-based activities were included, it would be interesting to evaluate to what extent other recreational-educational activities influence the observed reinforcement of resilience.

Given the results observed, which show how the school lessons in the hospital school can improve the resilience of hospitalised children and considering anxiety as the most common negative response to hospitalisation (Godino-Iáñez et al., 2020), it would be worth exploring whether the improvement in resilience is also associated with an improvement in the children’s tendency toward anxiety.

As hospital schools work heterogeneously, there is a lack of similar studies in other countries. For this reason, it would be of great interest to try and replicate our study in worldwide hospital schools.

### Limitations of the Study

Limitations of the sample size: Although significant results were obtained, the limited sample size of this study may require replicating the presented protocol in a larger cohort. In general, including children in hospital school studies differs from other educational settings. This is because inclusion in hospital settings is much more challenging due to the physical health status of the children and the psychological state of both the children and their parents.

Limitations of the resilience measurement interval: The theoretical limitation of the short interval of time spent in the hospital school in which the assessment of resilience was carried out is qualified by other studies that have demonstrated the effectiveness of programmes applied for one week, a similar time to that of this study.

Limitations of the assessment of the results: Having assessed resilience based on self-reports, it cannot be ruled out that there may be biases in the responses. In addition, the scarcity of experimental studies in this field may complicate the analysis of the results. Therefore, further studies are needed to contrast the obtained results.

Limitations related to other confounding factors: Factors such as family support have not been included in the present work. These aspects could be included in future lines of work to complement the current study.
